# Quantification of aortic stiffness across the cardiac cycle using magnetic resonance elastography

**DOI:** 10.1186/1532-429X-16-S1-P389

**Published:** 2014-01-16

**Authors:** William Kenyhercz, Anirudh Damughatla, Brian Raterman, Peter A Wassenaar, Richard D White, Arunark Kolipaka

**Affiliations:** 1 Radiology, The Ohio State University, Columbus, Ohio, USA; 2 Biomedical Engineering, The Ohio State University, Columbus, Ohio, USA; 3 Internal Medicine, The Ohio State University, Columbus, Ohio, USA

## Background

The measurement of arterial stiffness has long been a reliable method of determining the severity and risk involved with many cardiovascular diseases [1]. Magnetic resonance elastography (MRE), a non-invasive MRI-based technique, has recently been applied to measure aortic stiffness [2]. The aim of this study is to determine MRE-derived shear stiffness (μMRE) of the abdominal aorta over the cardiac cycle.

## Methods

**Figure 1:**
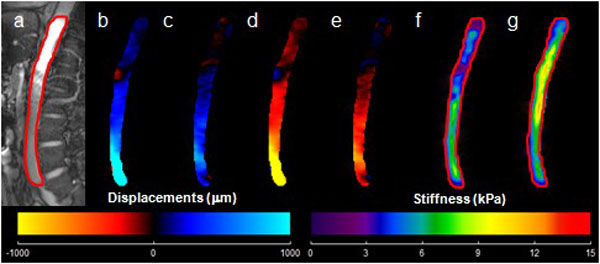
**(a): Sagittal magnitude image with contour (red) outlining aorta**. (b-e): Wave propagation at four points in time. (f, g): Weighted stiffness maps from x, y, and z encoding directions with a mean μMRE of 5.1 ± 1.4 kPa and 7.2 ± 1.6 kPa for end-diastole and end-systole respectively.

In-vivo aortic MRE was performed on 5 healthy volunteers ranging from 19-33 years of age. Imaging was performed on a 3T-MRI Scanner (Tim-Trio, Siemens Healthcare, Germany). Volunteers were positioned head first in the supine position in the scanner. 70 Hz mechanical waves were introduced into the aorta using a pneumatic driver system [2]. A 2D segmented, retrospective cardiac-gated, gradient-recalled echo MRE multi-slice cine sequence was used to acquire sagittal slices covering the abdominal aorta. The imaging parameters included: α = 25°, TE = 11.17-12.29, TR = 14.3 ms, FOV = 40 cm^2^, matrix = 128×64, slice thickness = 6 mm, #slices = 3, #segments = 7-8, 8 cardiac phases, and a motion encoding gradient (MEG) ranging from 90-100 Hz applied separately in the x, y, and z directions. Sagittal images were masked to delineate the abdominal aorta (Figure 1) for data analysis and MRE wave images were analyzed with MRE-Lab (Mayo Clinic, Rochester, MN) to yield the 3D stiffness (μMRE) values of the aortic tissue [3]. Standard bSSFP cine cardiac imaging was performed to determine trigger times for end-diastolic and end-systolic phase, which were then matched with data from the scan to determine stiffness at the aforementioned points in the cardiac cycle.

## Results

**Figure 2:**
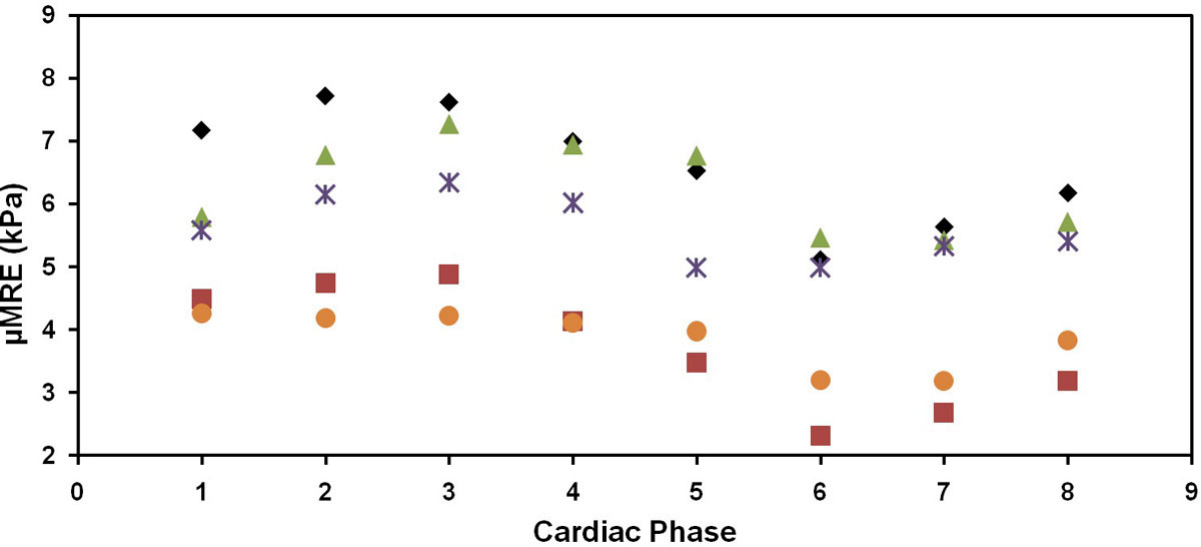
**Plot of MRE-derived stiffness (μMRE) throughout the cardiac cycle**. Stiffness values were higher around end systolic phase and lower around end diastolic phase.

Figure 1 shows a magnitude image with a red contour delineating the abdominal aorta in a volunteer (a); four images demonstrating the wave propagation through the aorta (b-e); and the MRE-weighted stiffness maps from three encoding directions for both end-diastolic phase (f) and end-systolic phase (g) using a local frequency estimation inversion algorithm. The mean stiffness values for end-diastolic and end-systolic phase shown in Figure 1 are 5.1 ± 1.4 kPa and 7.2 ± 1.6 kPa, respectively. Figure 2 shows a plot of μMRE at various points in the cardiac cycle for five volunteers with stiffness values higher around end-systolic phase and lower around end-diastolic phase.

## Conclusions

This preliminary study demonstrated the feasibility of determining aortic stiffness values during the cardiac cycle and also showed that aortic stiffness values varied during the cardiac cycle. The stiffness values are lower during the diastolic phases when compared to systolic phases. However, additional data is warranted to establish the stiffness values of the aorta during the cardiac cycle.

## Funding

The Ohio State University Department of Radiology.
